# Complete Genome Sequence of a thermotolerant sporogenic lactic acid bacterium, *Bacillus coagulans* strain 36D1

**DOI:** 10.4056/sigs.2365342

**Published:** 2011-12-22

**Authors:** Mun Su Rhee, Brélan E. Moritz, Gary Xie, T. Glavina del Rio, E. Dalin, H. Tice, D. Bruce, L. Goodwin, O. Chertkov, T. Brettin, C. Han, C. Detter, S. Pitluck, Miriam L. Land, Milind Patel, Mark Ou, Roberta Harbrucker, Lonnie O. Ingram, K. T. Shanmugam

**Affiliations:** 1 Department of Microbiology and Cell Science, University of Florida, Gainesville, FL, USA; 2 Bioscience Division, Los Alamos National Laboratory, Los Alamos, New Mexico, USA; 3 Joint Genome Institute, Walnut Creek, CA, USA; 4 Oak Ridge National Laboratory, Oak Ridge, TN, USA

**Keywords:** *Bacillus coagulans*, genome sequence, lactic acid, fermentation, probiotics, thermotolerant bacterium

## Abstract

*Bacillus coagulans* is a ubiquitous soil bacterium that grows at 50-55 °C and pH 5.0 and ferments various sugars that constitute plant biomass to L (+)-lactic acid. The ability of this sporogenic lactic acid bacterium to grow at 50-55 °C and pH 5.0 makes this organism an attractive microbial biocatalyst for production of optically pure lactic acid at industrial scale not only from glucose derived from cellulose but also from xylose, a major constituent of hemicellulose. This bacterium is also considered as a potential probiotic. Complete genome sequence of a representative strain, *B. coagulans* strain 36D1, is presented and discussed.

## Introduction

In addition to its use in food and cosmetics, lactic acid is increasingly used as a starting material for production of bio-based, renewable plastics [1-3]. Optically pure lactic acid required by the bioplastics industry is currently produced only by bacterial fermentation of sugars [3,4]. The main sugars currently used in such fermentations are glucose derived from corn starch or sucrose from sugar cane, sugar beets, etc. With increasing demand for renewable bio-based plastics, there is a shift away from food-based carbohydrates to non-food carbohydrates such as lignocellulosic biomass for lactic acid production [5,6]. Commercial fungal cellulases play a central role in the conversion of cellulose to glucose before fermentation to lactic acid and these enzymes function optimally at 50°C and pH 5.0 [7-10]. By matching the fungal enzyme activity optimum with that of the growth and fermentation optimum of the microbial biocatalyst, such as *Bacillus coagulans*, the amount of fungal cellulases required for simultaneous saccharification and fermentation (SSF) of cellulose to lactic acid can be reduced by a factor of three or higher compared to fermentation with lactic acid bacteria that grow optimally at temperatures below 40°C [9]. Since fungal enzymes represent a significant cost component of the overall process of biomass conversion to fuels and chemicals [11], reducing the enzyme loading during SSF of cellulose to lactic acid by *B. coagulans* is expected to lower the overall process cost and help the bioplastics industry compete with petroleum-based non-renewable plastics.

*Bacillus coagulans* belongs to a group of bacteria classified as sporogenic lactic acid bacteria [12]. These facultative anaerobes ferment pentoses, a component of hemicellulose, to L(+)-lactic acid as the major fermentation product reaching yields of 90% and titers close to 100 g/L in about 48 hours [13,14]. In this regard, *B. coagulans* differs from other lactic acid bacteria, such as *Lactobacillus, Lactococcus*, etc., in its ability to ferment pentose sugars to lactic acid through the pentose-phosphate pathway in contrast to the phosphoketolase pathway used by the lactic acid bacteria that yield an equimolar mixture of lactate and acetate [14]. Because of the thermotolerant, acid-tolerant and pentose fermentation characteristics, there is significant commercial interest in developing *B. coagulans* as a microbial biocatalyst for production of optically pure lactic acid as well as other fuels and chemicals. The higher operating temperature of *B. coagulans* is also expected to significantly reduce contamination of industrial fermentations that could lower product quality [15].

*B. coagulans* has been reported to function as a probiotic in animal trials and there is significant interest in the potential of this bacterium as a probiotic in humans [16]. These studies suggest that *B. coagulans* can readily achieve the GRAS (generally regarded as safe) status required for large scale industrial use. Genetic tools are being developed for manipulating *B. coagulans*, a genetically recalcitrant bacterium [17,18]. In order to fully explore the potential of *B. coagulans* as a microbial biocatalyst for production of fuels and chemicals, the entire genome of *B. coagulans* strain 36D1 was sequenced. Results from these experiments reveal that strain 36D1 has a single circular genome of 3,552,226 base pairs that encode 3,306 protein coding regions. Other characteristics of this bacterium, based on its genome composition, are presented and discussed.

## Classification and features

**Fig. 1:**
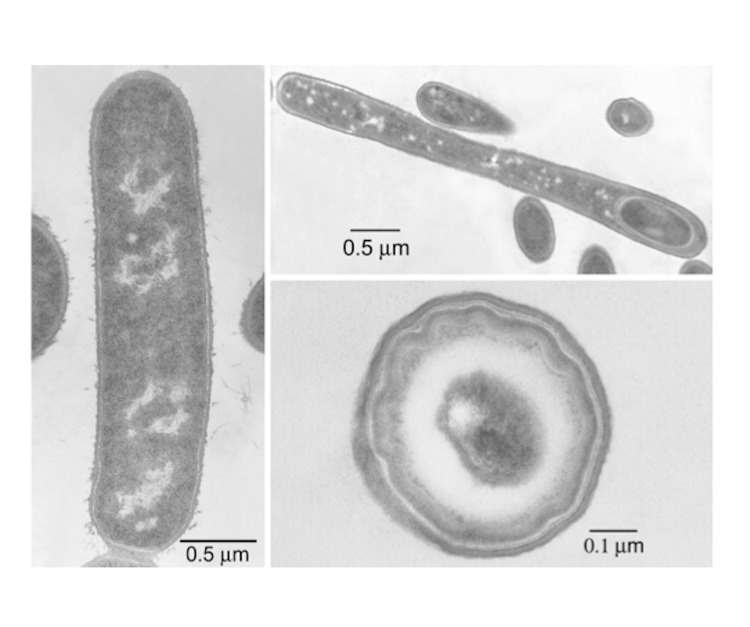
Thin section electron micrographs of *B. coagulans* strain 36D1. Left: vegetative cell. Upper right: sporulating cell. Lower right: mature spore.

*B. coagulans* was first isolated from coagulated milk by Hammer in 1915 [19]. Since then, several members of this group have been isolated from various sources [12,14]. *B. coagulans* strain 36D1 used in this study was isolated from a mud sample from an effluent stream of Old Faithful Geyser 1 near Calistoga, California, USA as an organism that can grow on xylose at 50°C and pH 5.0 both aerobically and anaerobically [14]. This bacterium is rod-shaped and produces endospore when cultured in nutrient broth (Fig. 1). Endospores are rarely observed when the bacterium was cultured in L-broth. Optimum temperature and pH for growth of strain 36D1 is 55°C and 5.5, respectively [10]. Corn steep liquor at 0.5% (w/v) provided the needed nutritional supplements for growth in mineral salts medium and the growth rate of the bacterium in that medium at 55°C was 1.67 h^-1^. The main fermentation product of the bacterium is L-lactate. Pentose fermentation increases the level of acetate, ethanol and formate in the medium compared to hexose fermentation [14]. Anaerobic cultures started with sparging of the medium with N_2_ require CO_2_ for growth. Other characteristics of the bacterium are listed in Table 1. *B. coagulans* strain 36D1 is deposited in the American Type Culture Collection (PTA-5827).

**Table 1:** Classification and general features of *Bacillus coagulans* strain 36D1

**MIGS ID**	**Property**	**Term**	**Evidence code**
MIGS-2	Classification	Domain *Bacteria*	TAS [20]
		Phylum *Firmicutes*	TAS [21-23]
		Class *Bacilli*	TAS [24,25]
		Order *Bacillales*	TAS [26,27]
		Family *Bacillaceae*	TAS [26,28]
		Genus *Bacillus*	TAS [26,29,30]
MIGS-3		Species *Bacillus coagulans*	TAS [12,19,26]
		Strain 36D1	
	Gram stain	Positive	IDA
	Cell shape	Rods	IDA
	Motility	Yes	IDA
	Sporulation	Yes	IDA
	Temperature range	Thermotolerant; 30 – 60 °C	TAS [10]
	Optimum temperature	55 °C	TAS [10]
			
			
			
			
			
MIGS-22	Oxygen requirement	Facultative anaerobe	TAS [14]
			
	Carbon source	Glucose, xylose, arabinose, galactose, maltose, fructose, cellobiose, yeast extract, peptone	IDA
			
	Energy source	Organic carbon compounds	IDA
	Terminal electron acceptor	O_2_, acetate, pyruvate	TAS [14]
MIGS-6	Habitat	Soil	TAS [14]
MIGS-15	Biotic relationship	Free-living	TAS [14]
MIGS-14	Pathogenicity	None; potential probiotic	NAS
	Biosafety level	1	NAS
	Isolation	Soil	TAS [14]
MIGS-4	Geographic location	Near Calistoga, California, USA	TAS [14]
MIGS-5	Sample collection time	2000	IDA
MIGS-4.1	Latitude	38.58 N	NAS
MIGS-4.2	Longitude	-122.58	NAS
MIGS-4.3	Depth	Surface	TAS [14]
MIGS-4.4	Altitude	351’	NAS

TAS, traceable author statement; IDA, inferred from direct assay; NAS, non-traceable author statement. These evidence codes are from the gene ontology project [31].

**Fig. 2:**
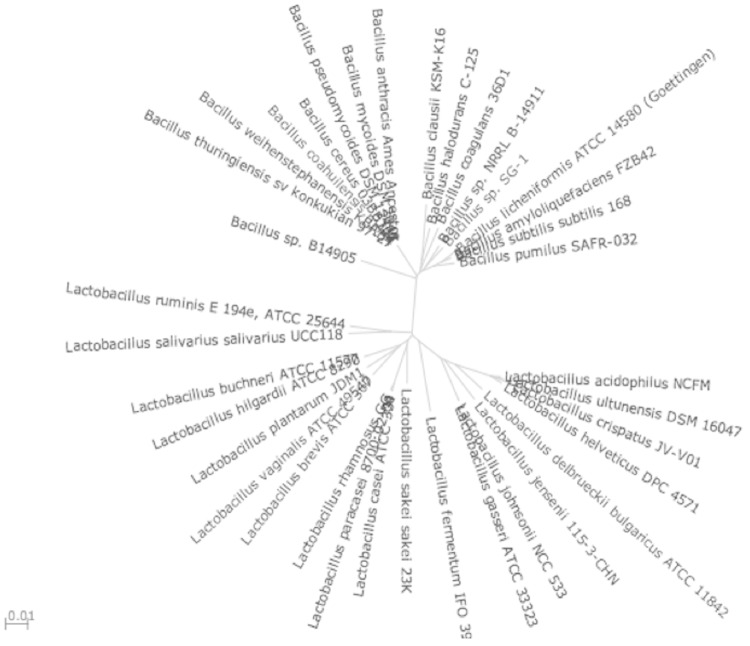
Unrooted phylogenetic tree based on 16S rRNA(DNA) of *B. coagulans* strain 36D1 and *Bacillus* spp. and *Lactobacillus* spp.

The *B. coagulans* group is polydisperse [12] and among the *Bacillus* spp., strain 36D1 is phylogenetically close to *B. halodurans* based on 16S rRNA(DNA) sequences (Fig. 2). Although *B. coagulans* is similar to lactic acid bacteria in its ability to grow anaerobically and ferment sugars to lactic acid, it is distinct from the lactic acid bacteria based on 16S rRNA(DNA) sequence similarity.

## Genome sequencing and annotation

### Genome project history

This genome was selected for sequencing on the basis of the properties described above. The genome sequence is deposited in GenBank (Accession number, CP003056). Sequencing was initiated and completed to a level of four contigs and annotated by the DOE Joint Genome Institute (JGI). The original draft version was deposited in GenBank on February 7, 2007 and the final draft version with four contigs was deposited on Feb. 3, 2010, thereby updating previous releases to the database. Genome sequencing was completed at the University of Florida, annotated by the Oak Ridge National Laboratory, and processed by the Los Alamos National Laboratory and NCBI. A summary of the project information is shown in Table 2.

**Table 2:** Genome sequencing project information

**MIGS ID**	**Property**	**Term**
MIGS-31	Finishing quality	Finished
MIGS-28	Libraries used	Genomic libraries: Sanger (one each of 2.5, 8 and 37 kbp library); one 454CT library (2 kb); PCR; primer walk
MIGS-29	Sequencing platforms	Sanger; 454
MIGS-31.2	Sequencing coverage	9×
MIGS-30	Assemblers	Phrap, Newbler
MIGS-32	Gene calling method	Prodigal
	Genome Database	GenBank
	INSDC ID	CP003056
	Genbank Date of Release	September 26, 2011
	GOLD ID	GC01988
	NCBI project ID	15679
MIGS-13	Source material identifier	ATCC PTA-5827
	Project relevance	Biotechnological

### Growth conditions and DNA isolation

*B. coagulans* strain 36D1 was cultured in LB + glucose (10 g/L) medium (pH 5.0) at 50°C in a shaker at 200 RPM as described before [10]. Cells were harvested during mid-exponential phase of growth. Cell pellet from a 30 ml culture was resuspended in 2.1 ml of TE buffer (Tris, 10 mM; EDTA, 10 mM; pH 8.0) supplemented with lysozyme (1 mg/ml; Sigma Chemical Co., St. Louis, MO, USA) and RNase (0.1 mg/ml; Sigma Chemical Co.). The sample was incubated at 37°C for 20 minutes to remove the cell wall. Sodium dodecyl sulfate (SDS) was added to the lysed cells to achieve an SDS concentration of 1.4%. After 10 minutes on ice, the lysate was extracted with equal volume of TE-saturated phenol to remove cellular debris. After two more extractions of the aqueous phase with equal volumes of phenol-chloroform mixture (25:24:1 of phenol, chloroform and isoamyl alcohol), and one extraction with an equal volume of chloroform:isoamyl alcohol, the DNA was precipitated with ethanol and dried. The ratio of absorbance at 260 nm and 280 nm of the purified DNA was 1.99 and based on agarose gel electrophoresis and ethidium bromide staining, DNA contained only a trace amount of degraded RNA.

### Genome sequencing and assembly

The genome was sequenced using a combination of Sanger and 454 sequencing platforms. General aspects of library construction and sequencing can be found at the JGI website [32]. 454 pyrosequencing reads were assembled using the Newbler assembler version 1.1.02.15 (Roche). Large Newbler contigs were broken into 2 kb overlapping fragments (1 kb overlap) and entered into assembly as pseudo-reads. The sequences were assigned quality scores based on Newbler consensus q-scores with modifications to account for overlap redundancy and to adjust inflated q-scores. A hybrid 454/Sanger assembly was made using the Phrap assembler. Possible mis-assemblies were corrected with Dupfinisher or transposon bombing of bridging clones. Editing in Consed, custom primer walk or PCR amplification closed gaps between contigs. A total of 2,471 Sanger finishing reads were produced to close gaps, to resolve repetitive regions, and to raise the quality of the finished sequence. The error rate of the completed genome sequence was less than 1 in 100,000. Together all sequence types provided 9 x coverage of the genome. The final assembly contains a total of 35,357 Sanger and pyrosequence reads. This analysis yielded four contigs with lengths of 2,712, 65,471, 565,365 and 2,917,758 base pairs for a total of 3,551,306 base pairs.

In order to close the gaps, a restriction map of *B. coagulans* strain 36D1 genome was constructed using BglII restriction enzyme. This optical mapping by OpGen (Gaithersburg, MD) yielded a circular map of approximately 3,521 kbp. Comparing the computed restriction map of the DNA sequence from the four contigs with the restriction map of the whole genome, the lengths of the gaps between the appropriate contigs were predicted. Using the sequence information from the contigs and appropriate restriction fragments, PCR primers were synthesized and the genomic DNA was sequenced using Sanger method by the Interdisciplinary Center for Biotechnology Research at the University of Florida. As needed, PCR primers were synthesized based on new sequence information for genome walking to fill-in the gaps and complete the genome sequence. Based on these analyses, the genome of *B. coagulans* strain 36D1 was determined to be circular with a length of 3,552,226 base pairs.

### Genome annotation

Genes were identified using Prodigal [33] as part of the Oak Ridge National Laboratory genome annotation pipeline. The predicted CDSs were translated and used to search the National Center for Biotechnology Information (NCBI) nonredundant database, UniProt, TIGRFam, Pfam, PRIAM, KEGG, COG, and InterPro databases. These data sources were combined to assert a product description for each predicted protein. Non-coding genes and miscellaneous features were predicted using tRNAscan-SE [34], RNAMMer [35], Rfam [36], TMHMM [37], and signalP [38].

## Genome properties

**Fig. 3:**
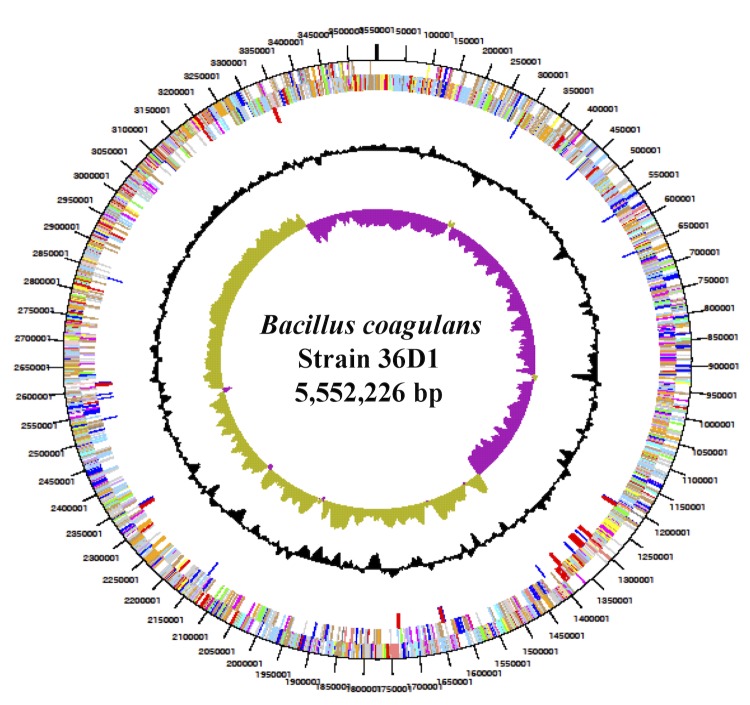
Graphical circular map of the genome of *B. coagulans* strain 36D1. From outside to center: Genes on forward strand (color by COG categories), Genes on reverse strand (color by COG categories), pseudogenes, % G+C, GC skew.

The genome consists of a 3,552,226 bp long chromosome with a 46.5% GC content (Table 3, Fig. 3). Of the 3,420 genes predicted, 3,306 were protein coding genes, and 114 encode RNAs. Among the 114 RNA genes, 10 each coded for 5S, 16S and 23S rRNAs and 84 can be accounted for tRNAs.

**Table 3:** Genome statistics

**Attribute**	**Value**	**% of total**
Genome Size (bp)	3,552,226	100
DNA G+C content (bp)	1,651,327	46.5
Number of replicons	1	
Extrachromosomal elements	0	
Total genes	3420	100
RNA genes	114	3.3
Protein-coding genes	3306	96.7
Genes in paralog clusters	541	15.6
Genes assigned to COGs	2456	71.0
Genes with signal peptides	858	24.8
Genes with transmembrane helices	863	24.9
Paralogous groups	199	5.8

**Fig. 4:**
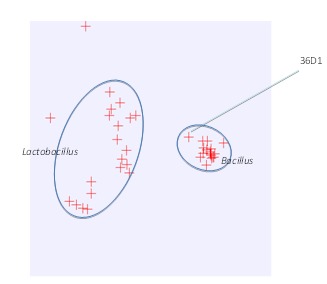
Principal component analysis based on COG profiles

The majority of the protein-coding genes (74%) were assigned with a putative function while those remaining were annotated as hypothetical proteins. About 49 ORFs were identified as potential transposases. The distribution of genes into COGs functional categories is presented in Table 4. The first about 40% of the genome is predominantly transcribed from the lagging strand (as written) while the other 60% is transcribed from the leading strand (Fig. 4).

**Table 4:** Number of genes associated with the general COG functional groups

**Code**	**Value**	**%age**	**Description**
J	172	5.14	Translation
A	0	0.00	RNA processing and modification
K	238	7.11	Transcription
L	144	4.30	Replication, recombination and repair
B	2	0.05	Chromatin structure and dynamics
D	106	3.16	Cell cycle control, mitosis and meiosis
Y	0	0.00	Nuclear structure
V	113	3.37	Defense mechanisms
T	162	4.84	Signal transduction mechanisms
M	240	7.17	Cell wall/membrane biogenesis
N	78	2.33	Cell motility
Z	0	0.00	Cytoskeleton
W	0	0.00	Extracellular structures
U	50	1.49	Intracellular trafficking and secretion
O	182	5.44	Posttranslational modification, protein turnover, chaperones
C	277	8.28	Energy production and conversion
G	310	9.26	Carbohydrate transport and metabolism
E	379	11.33	Amino acid transport and metabolism
F	100	2.98	Nucleotide transport and metabolism
H	217	6.48	Coenzyme transport and metabolism
I	104	3.10	Lipid transport and metabolism
P	214	6.39	Inorganic ion transport and metabolism
Q	168	5.02	Secondary metabolites biosynthesis, transport and catabolism
R	448	13.39	General function prediction only
S	242	7.23	Function unknown
-			Not in COGs (Total – 3345)

## Insights from genome sequence

Comparison of the predicted proteome of *B. coagulans* with that of a group of *Bacillus* spp genomes identified 491 unique proteins in *B. coagulans* that are not identified in other members of *Bacillus* spp. 404 of these genes are in the early part of the 36D1 genome as listed. This list includes 31 genes encoding putative transposases. Function of many of these gene products is not known. However, 413 of these unique proteins are found to be shared with *Lactobacillus* spp. In reverse, comparison of the *B. coagulans* genome with the genomes from a group of *Lactobacillus* spp. revealed that 423 ORFs are unique to *B. coagulans* and of these, 345 ORFs, mostly related to sporulation, are shared with *Bacillus* spp. Combining these two sets, a set of 78 ORFs coding for proteins with unknown function are unique to *B. coagulans* that are not present in either *Bacillus* or *Lactobacillus*. Based on principal components analysis, *B. coagulans* strain 36D1 groups with *Bacillus* but as an outlier and away from Lactic acid bacteria (Fig. 4). Although *B. coagulans* produced L-lactic acid as the fermentation product at an optical purity reaching close to 100%, the genome contains a gene encoding D-LDH.

Although some members of *B. coagulans* group are cellulolytic and xylanolytic, strain 36D1 is phenotypically unable to utilize cellulose and xylan. However, genes encoding glycan hydrolases such as xylanase, xylosidase and α-amylase can be identified in the genome sequence. Presence of these genes suggest that the bacterium can be evolved to produce xylanase to reduce the severity of acid treatment during hydrolysis of hemicellulose from lignocellulosic biomass for production of optically pure lactic acid. *B. coagulans* strain 36D1 is an auxotroph for several amino acids and vitamins. Based on analysis of the genome sequence by Patric Comparative pathway tool [39], only histidine biosynthetic pathway appears to be incomplete among the amino acid biosynthesis pathways. Among the vitamins, the pathways for biosynthesis of biotin, pantothenic acid, nicotinamide and pyridoxine appear to be incomplete.

During the time of preparation of this manuscript, genome sequence for *B. coagulans* strain 2-6 was published [40]. The genome of this strain is 3,073,079 and is 479,147 bp smaller than the genome of strain 36D1. These two *B. coagulans* genomes share about 90% or higher nucleotide sequence identity in the regions that are present in both genomes. Additional comparative analysis of the two genomes is in progress.
